# Implementation of a Multisectoral Approach to Address Adolescent Pregnancy: A Case Study of Subnational Advocacy Informing National Scale-Up in Kenya

**DOI:** 10.9745/GHSP-D-22-00546

**Published:** 2023-10-30

**Authors:** Beatrice Kwachi, Sally Njiri, Sam Mulyanga, Irene Choge, Rammah Mwalimu, Susan Ontiri

**Affiliations:** a Advance Family Planning, Jhpiego Kenya, Nairobi, Kenya.; b International Center for Reproductive Health-Kenya, Mombasa, Kenya.

## Abstract

Adolescent pregnancy continues to be a major concern globally, especially in sub-Saharan Africa, where it contributes to high maternal mortality and morbidity and other non-health consequences, such as school dropouts. Addressing adolescent sexual and reproductive health requires an enabling environment that extends beyond an individual to community and society levels through concerted efforts by various sectors and agencies. From 2016 to 2022, we implemented an advocacy intervention in Kenya that catalyzed multisectoral collaboration to address adolescent sexual and reproductive health issues, specifically adolescent pregnancy, at subnational and national levels. In this article, we document our experiences implementing the intervention by presenting a case study of this initiative, including the processes and lessons learned. Our experience to date demonstrates that multisectoral initiatives can be rolled out to address adolescent pregnancies in countries that continue to have a high burden of adolescent pregnancy. Successful implementation requires inclusivity, commitment, and leadership to fully realize the potential that multisectoral actions have in preventing adolescent pregnancy.

## Abstract (precis)

Implementation of a multisectoral initiative to address adolescent pregnancy in Kenya required strong leadership and ownership at both national and subnational levels. Advocacy is key to harnessing leadership, ownership, and scale-up.

## Teaser

**Key Findings:**

- Implementation of a multisectoral initiative to address adolescent pregnancy in Kenya required strong leadership and ownership both at national and subnational levels.
- A multisectoral action plan allowed consensus on priorities while ensuring that the strategic interests of all stakeholders were maintained.
- Localization of adolescent pregnancy data at various levels of decision-making enabled evidence-based advocacy, localized challenges, and triggered action.

**Key Implications:**

- Successful implementation of a multisectoral approach requires inclusivity, commitment, and leadership to fully realize the potential that multisectoral actions have in addressing adolescent pregnancy, health, and well-being.
- Advocacy is key in driving multisectoral initiatives aimed at addressing adolescent pregnancies.

## BACKGROUND

Adolescent pregnancy, defined as the occurrence of pregnancy in girls aged 10–19 years, continues to be a global phenomenon, especially in sub-Saharan Africa, where the prevalence is 19.3%.^1^^–^^3^ Studies have shown that adolescent pregnancy increases the risk of maternal and child mortality compared to pregnancy among older women.^4^ Indeed, complications relating to pregnancy and childbirth have been noted as the leading cause of death for adolescent girls (aged 15–19 years) globally.^4^^,^^5^ The risk goes beyond health, as it has a devastating effect on individual girls when dreams are shattered or deferred due to increased economic and social burden, which is further compounded by social ostracization.^6^ Consequently, this ultimately affects the socioeconomic status of a country as pregnant adolescents are more likely to drop out of school, further perpetuating the cycle of poverty.^7^^,^^8^

Addressing adolescent sexual and reproductive health (ASRH) requires an enabling environment that extends beyond an individual to community and societal levels.^9^ For improved outcomes, it is widely acknowledged that multisectoral actions in health that span various ministries, government agencies, development sectors, and other stakeholders are needed to address existing and emerging issues.^10^ However, there is a lack of evidence of an effective implementation approach and outcomes from multisectoral collaboration and integration.^11^

Adolescents' sexual and reproductive health needs continue to be unmet due to various factors, including lack of knowledge, attitude, stigma, and restrictive and punitive laws.^12^ The World Health Organization recognizes that achieving optimal health and well-being for adolescents requires adoption of multisector approaches and effective intersectoral coordination and collaboration.^13^ Although there is much literature on the importance of multisectoral adolescent health interventions, limited information exists on how they can be implemented to address ASRH globally and in sub-Saharan Africa, specifically.^10^ Whereas the benefits of multisectoral collaboration for adolescent health are not debatable, there is a general lack of information on how to implement collaboration initiatives, considering that this approach is complex and dependent on many crucial considerations, such as leadership and political will, resources, coordination, and communication.^14^ For example, only 3 of the 12 case studies published in a 2019 *BMJ* special series on multisectoral collaboration in maternal, child, and adolescent health focused on adolescent health—2 on nutrition and 1 on HIV.^15^ In today's interconnected and interdependent world, examples of multisectoral approaches to ASRH that have been implemented need to be shared to show how collaborative approaches can foster inclusivity and ownership to enhance efficiency and sustainability of solutions. Against this backdrop, we document our experience implementing a multisectoral collaboration initiative in Kenya that addresses adolescent pregnancy. This case study describes the processes followed in establishing, implementing, monitoring, and scaling up the initiative and the lessons learned during the process.

Examples of multisectoral approaches to ASRH that have been implemented need to be shared to show how collaborative approaches can foster inclusivity and ownership to enhance efficiency and sustainability of solutions.

## MULTISECTORAL COLLABORATION INITIATIVE DESCRIPTION

The Advance Family Planning (AFP) initiative (2009–2022) comprised multiple organizations worldwide working to expand access to voluntary, quality contraceptive information and services by securing an enabling environment that increases financial investments and political commitments. This was achieved through an evidence-based, SMART (specific, measurable, attainable, relevant, and time-bound) Advocacy approach. SMART Advocacy is a guide to developing advocacy strategies that lead to quick wins by reaching the right decision-maker with the right message at the right time.^16^

In Kenya, the AFP initiative was implemented by Jhpiego, with funding from the Bill & Melinda Gates Institute for Population and Reproductive Health, across the national and subnational levels. Kenya has a devolved form of government where power has been transferred from national level to county level. The national-level leadership remains with the President of Kenya, and each of the 47 counties has an elected governor.^17^ There is a clear separation of power and mandate across the 2 levels.^17^ For example, under the national Ministry of Health, the national government is tasked with the development of policies and guidelines, while at the subnational level, county departments of health are mandated with implementation and contextualization.^17^ Since the AFP's inception in Kenya, the advocacy approach at the county level involved working with the County First Ladies Association—a platform that brings together spouses of governors from all 47 counties in Kenya—to secure an enabling environment for family planning.

Findings from the 2014 Kenya Demographic and Health Survey revealed that almost 1 of 5 (18%) adolescent girls aged 15–19 years were either pregnant or had a live birth.^18^ The survey findings further showed that the prevalence of adolescent pregnancy in the 47 counties ranged from 6% to 40%.^18^ Based on these results, the initiative added a component of ASRH as part of its intervention in 2016. The initiative conducted an ASRH landscape analysis to assess existing interventions and gaps. One of the emerging findings was that even though Kenya's ASRH policy acknowledged the need for multisectoral or intersectoral collaboration,^16^ there had been limited efforts to coordinate programs. In response, the initiative decided to focus its ASRH intervention on establishing and implementing a multisectoral approach based on the evidence-based SMART Advocacy approach that was being used in the broader initiative's thematic areas of family planning, including finance, media advocacy, private-sector engagement, and task-sharing.

## HOW MULTISECTORAL COLLABORATION WAS ACHIEVED

**FIGURE 1:**
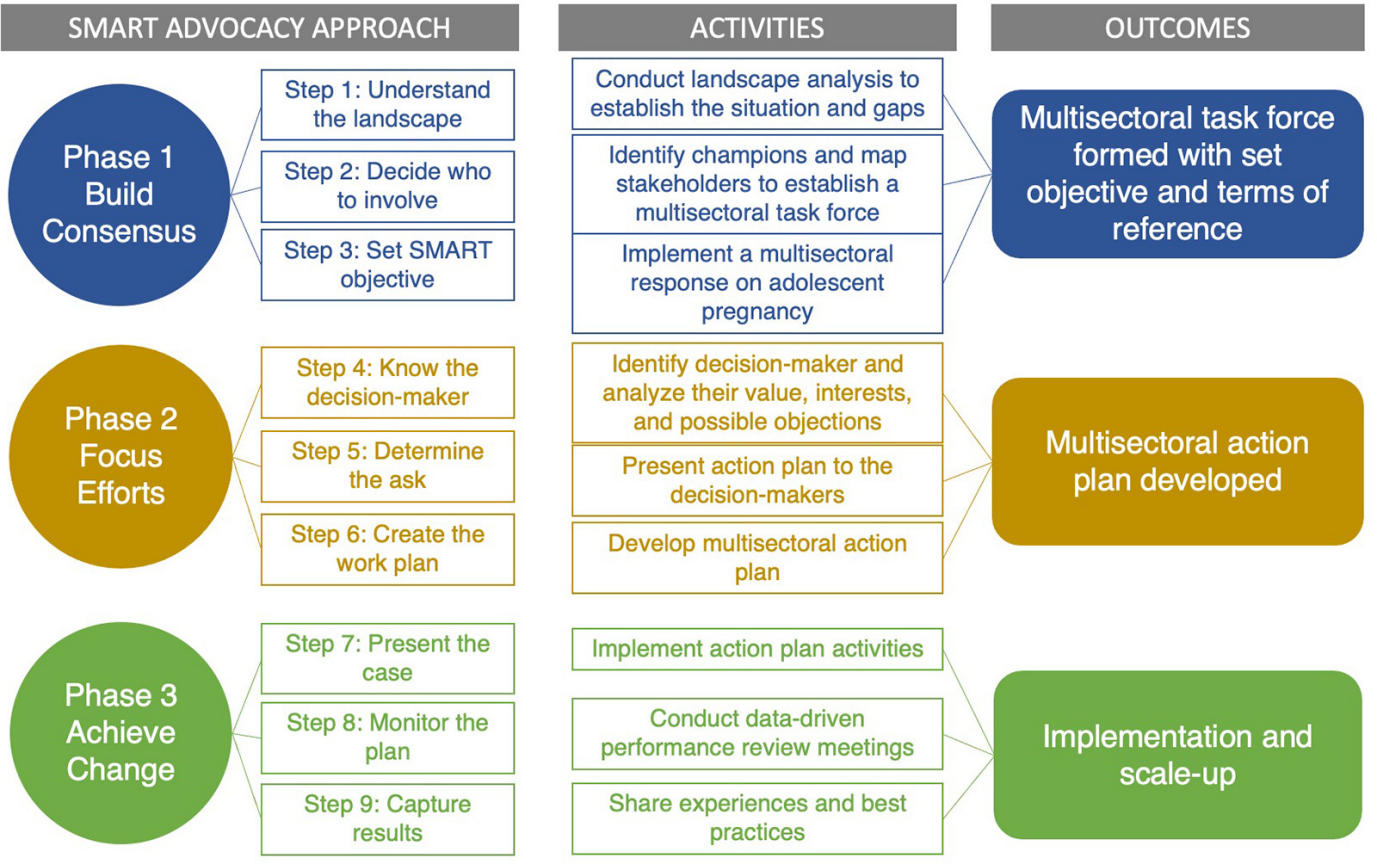
Implementation Process of Multisectoral Initiative in Kenya Through SMART Advocacy Approach Abbreviation: SMART, specific, measurable, achievable, relevant, and time bound.

The multisectoral intervention was implemented in 3 phases (Figure 1). Phase 1 entailed establishing multisectoral collaboration; phase 2, operationalizing the multisectoral task force; and phase 3, scaling up the multisectoral approach.

### Phase 1: Establishment of Multisectoral Collaboration

#### Building of Strategic Partnerships

A key learning from the landscape analysis was that, whereas several ASRH interventions were being implemented across the country, there was a lack of coordinated efforts across various sectors, which often led to duplication and lack of constructive collaboration. The analysis identified interventions and key actors working on adolescent pregnancy prevention and ASRH. The key actors, both at county and national levels, included government ministries and/or departments of health, education, interior, gender, and youth and children services, as well as the judiciary. Other actors included faith-based organizations, community cultural gatekeepers, youth-led organizations, and implementing partners largely drawn from civil society.

The landscape analysis showed that several ASRH interventions were being implemented across Kenya, but there was a lack of coordinated efforts across various sectors, often leading to duplication and lack of constructive collaboration.

The initiative also leveraged the pool of county- and national-level journalists that AFP had trained as part of media advocacy. These journalists were championing family planning in the media by telling stories and holding leaders and decision-makers accountable for their actions. Details of media advocacy conducted by the trained journalists are published elsewhere.^19^

To foster intersectoral collaboration, we identified the strategic interest of each of the key actors identified in the landscape analysis. For example, county departments of health reported a high number of adolescents presenting with pregnancy in health facilities. The departments of education, on the other hand, reported experiencing school dropouts, absenteeism, and poor performance because of pregnancy among adolescents.

Information on the strategic interest of each entity was used to develop targeted messages when soliciting their buy-in. The initiative liaised with like-minded stakeholders to facilitate meetings that brought together key actors to share their interest in the prevention of adolescent pregnancy, agree on a common outcome, and commit to a multisectoral approach.

#### Identification of Local Champions to Spearhead the Multisectoral Initiative

The landscape analysis revealed the need for a unifying approach to bring all sectors together. Based on the insights gathered from stakeholders, we determined that county first ladies would be an appropriate group to help bring the sectors together, as their role spans various sectors. This was also informed by a similar approach taken at the national level, where Kenya's First Lady at the time of implementation, Margaret Kenyatta, identified maternal health as her priority area and launched the Beyond Zero campaign, which received support from all key stakeholders.^20^ Similarly, our landscape analysis revealed that activities conducted by the offices of the county first lady were perceived by various county government departments to align with the county governor's vision and harness political goodwill, which is essential in creating a favorable environment for ASRH interventions at the county level and for coordinating the various government ministries.

Leveraging the existing advocacy platform AFP had initiated in 2015 with the County First Ladies Association, the AFP team contacted the chair of the association and expressed an interest in partnering with them. Consequently, the initiative convened a consultative meeting with the association. Through their first ladies, 3 counties (Narok, Kwale, and Busia) agreed to pioneer the implementation of multisectoral action in the first quarter of 2017. The county first ladies felt a pressing need to address adolescent pregnancy because of the high burden in those counties—Narok, 40%; Kwale, 24%; and Busia, 21%—compared to the national average of 18%.^18^ The 3 counties are in different geographical regions: Narok in Rift Valley, Kwale in Coastal, and Busia in Western. The willingness of the county first ladies to become champions of ASRH provided the impetus needed to roll out the multisectoral initiative in their respective counties.

The willingness of the county first ladies to become champions of ASRH provided the impetus needed to roll out the multisectoral initiative in their respective counties.

#### Formation of a Multisectoral Task Force

Between April and June 2017, the 3 county first ladies contacted the various key actors within and outside of their county governments. The offices of the county first ladies also liaised with the leadership of the various departments at the county level to attend the first multisectoral meeting on adolescent pregnancy, which they each helped convene in their own county with financial support from the initiative. This was a critical step that explored and charted the path toward the establishment of county multisectoral task forces on the prevention of adolescent pregnancy. The meeting convened officers from the departments of health, education, youth, gender affairs, interior, and judiciary who had been selected by their respective leaders to form the task force. In each of the counties, the task forces were later expanded to include nongovernment entities, such as civil society organizations, youth, and religious and community leaders. The task forces provided a shared vision for accelerating the prevention of adolescent pregnancy through a multisectoral approach. Each county task force developed its terms of reference that clearly outlined roles and responsibilities (Box). The county first ladies undertook the role of patron for the task force.

BOX Terms of Reference for County Multisectoral Task Forces to Address Adolescent Pregnancy in Kenya

- Streamline the coordination of adolescent pregnancy interventions
- Develop a joint multisectoral action plan
- Coordinate reporting of adolescent pregnancy data
- Mobilize resources
- Convene progress reviews to assess the implementation of action plans
- Provide clarity on roles and responsibilities for each entity and the activities to be implemented by each institution

### Phase 2: Operationalization of Multisectoral Task Force

#### Development of a Joint Annual County Action Plan to Enhance Coordination

From July to September 2017, the initiative convened and financed county-level stakeholder meetings to develop joint multisectoral action plans. In addition to addressing adolescent pregnancy, the action plans also addressed related issues, such as school dropout rates and implementation of policies on school reentry and retention. The 3 counties used different approaches to develop their individual plans based on their context. For example, the Narok County action plan detailed activities that needed to happen in each of the subcounties. Kwale County identified schools with high adolescent pregnancy rates and developed an action plan based on data to address the issue within the schools' catchment areas.

These were the first action plans developed by county governments that clearly stipulated how the various agencies/departments would address ASRH. Development of the joint action plans provided an opportunity for stakeholders to identify their key thematic areas and reinforced their commitment to the implementation process by providing technical and financial support. The main guiding principle in the development process was collaboration. For example, to strengthen referral systems, the county departments of education linked schools with their nearest health facilities for the provision of ASRH information and services. Consequently, the departments of health planned to conduct outreaches in the schools and build the capacity of health workers on adolescent-friendly services. Table 1 outlines the specific roles of each stakeholder in the task force.

**TABLE 1.** Stakeholder Roles in Implementation of Adolescent Pregnancy Action Plan in Kenya

Stakeholder	Roles and Responsibilities
County department of health	Provide health services and information Strengthen the referral system between health facilities and learning institutions Build capacity of health workers Provide health data for decision-making
County department of education	Provide data on school dropouts and pregnancies Implement school reentry and retention policies for pregnant adolescents and those who have given birth Provide appropriate ASRH information by linking adolescents to health facilities
Ministry of Interior and Coordination	Coordinate and operationalize county multisectoral task forces Implement policies to address harmful cultural practices Enforce school reentry and retention policies for pregnant adolescents Reinforce law and order for defilement cases
Departments of children, youth, and gender	Link young people, including adolescent mothers, with economic empowerment opportunities Provide rescue and redress support services to adolescent and child survivors
Implementing partners	Mobilize and leverage resources Advocate for policy formulation and implementation Provide ASRH technical assistance
Youth-led organizations	Ensure meaningful youth (aged 10–24 years) participation in the implementation of the action plan Build capacity of youth advocates Advocate for improved ASRH services and information
Media establishments (radio and broadcast)	Generate public discussions on adolescent pregnancy and ASRH Hold decision-makers accountable for prevention of adolescent pregnancy Provide platform for ASRH information and catalyzing action on adolescent pregnancy
Parents and religious, cultural, and community leaders	Implement programs that empower adolescents and young people on their ASRH using their platforms Catalyze action against harmful practices at community level

Abbreviation: ASRH, adolescent sexual and reproductive health.

#### Meaningful Youth Engagement in Priority Setting

In the 3 counties (Narok, Kwale, and Busia), adolescents were involved in the entire process because they were part of the multisectoral task force. Participants from the youth-led organization included young people aged 18–25 years who shared their experiences. Each county used a different strategy to ensure meaningful participation of youth during planning and implementation, which was achieved through engagement with youth-led organizations at subnational levels. For example, in Narok County, youth who participated in the process were drawn from subnational youth technical working groups,youth councils, and youth-serving organizations. These young people brought technical experience in implementing youth-led initiatives, including achievements, lessons learned, and challenges.

#### Leveraging of Resources

To avoid duplication, government departments and partners mapped available resources by identifying partners' support. Departments were able to systematically assess the use of existing resources and identify the need for additional resources or new resources to address identified needs (e.g., leveraging existing funding streams or newly identified funding from agencies or organizations). The landscape analysis revealed that substantive resources were indirectly invested in ASRH across the sectors. However, no dedicated funding existed; hence, strategic planning and advocacy were necessary to ensure that existing investments would help to achieve the objectives. Stakeholders agreed that coming together would promote coordination and leverage existing financial and human resources.

The task force discussed financing of the county action plans' activities; consequently, partners were able to leverage each other's resources. Through this process, the counties were also able to identify activities they could support and ensure that funds were allocated for those activities. For example, in Kwale County, the health management team took up the role of convening performance review meetings and consequently allocated county resources to finance the meetings. In addition to leveraging financial resources, different technical expertise was acknowledged; in the action plans, the task force deliberately mapped the skillsets required to implement certain activities, which led to enhanced collaboration. These activities included the Department of Health building the ASRH capacity of the Ministry of Education team, including teachers.

#### Monitoring of Implementation Progress

The landscape assessment found that different sectors were addressing ASRH issues separately and that there was a lack of data sharing as no platform existed where different agencies could discuss ASRH data. It was evident that the health sector, by virtue of its mandate, had an established platform for data collection; hence, the task force relied on performance data shared by the departments of health. ASRH indicators that departments of health were already collecting included the number and proportion of adolescents seeking contraceptive services in facilities and the number of adolescents disaggregated by age (10–14 years and 15–19 years) presenting with pregnancy in facilities (first antenatal care [ANC] visit). In each of the performance review meetings, the Ministry of Health would share indicator data to show implementation progress.

### Phase 3: Scale-Up of the Multisectoral Approach

As the 3 counties were implementing their multisectoral approaches, the initiative and other partners continued deliberations across other counties and at the national level on the burden of adolescent pregnancy using existing platforms such as technical working group meetings. The multisectoral approach was scaled up, as detailed in the following sections.

#### Subnational Scale-Up

In the last quarter of 2018, the 3 county first ladies shared their experiences in catalyzing the formation and implementation of multisectoral initiatives in their respective counties with their peers during County First Ladies' Association forums, sparking an interest in additional counties. Based on the lessons learned, high-level leadership commitments, and support from the initiative, the multisectoral approach was expanded from the 3 initial counties (Narok, Kwale, and Busia) to 5 additional counties (Homa Bay, Migori, Kakamega, Kisii, and Siaya). Using the same approach and learning from the 3 initial counties, the 5 additional counties set up their multisectoral task forces and began implementation a year after the roll-out in the initial counties. The 5 additional counties received support from the initiative because of their high burden of adolescent pregnancy (Homa Bay, 33%; Migori, 24%; Kakamega, 20%; Kisii, 18%; and Siaya, 17%) and the commitment by the county leadership to implement the multisectoral initiative.

Based on the lessons learned, high-level leadership commitments, and support from the initiative, the multisectoral approach was expanded from the 3 initial counties to 5 additional counties.

Due to the success of the implementation model in Kwale County in terms of enhanced synergy in implementation of activities, officials from other counties, such as Migori, visited Kwale County to learn how they had successfully rolled out the multisectoral initiative. The objective of the learning visit was to identify areas for improvement, as Migori County was in the formative stage of its implementation. Based on these learnings, Migori County was able to form its multisectoral task force, albeit with some differences. For example, the process was driven by the Department of Health and not the County's First Lady. By the end of 2019, 8 counties were implementing the multisectoral approach. The approach enabled effective coordination across sectors, including improved monitoring from 3 different ministries—education, health, and interior—for decision-making and action planning.

#### Remodeling of the Multisectoral Approach During Expansion: Monitoring

Implementation of multisectoral collaboration in the 3 initial counties took an adaptive approach based on lessons learned during the start-up. In the scale-up to 5 additional counties, several changes were made to strengthen the role of each sector. One major revision was reducing the overreliance on health data in assessing the burden of adolescent pregnancy. In the scale-up counties, the task forces jointly developed a monitoring measurement framework that outlined (1) process and outcome indicators that task forces would monitor, (2) source of data, and (3) frequency for review. The monitoring and evaluation framework included process indicators that monitored collaboration to track its functionality as a form of accountability. Table 2 outlines the indicators by sector that were agreed upon for the monitoring plan. The task forces also developed a data capture tool that allowed each sector to collect more nuanced data on adolescent pregnancy or related areas.

**TABLE 2.** Indicators Selected for Monitoring Adolescent Sexual and Reproductive Health Interventions in Kenya

**Department of Health**	Number and proportion of pregnant women accessing antenatal care services who are adolescents aged 10–19 years Number of pregnant adolescents referred to health services Number of adolescents seeking contraceptive services Proportion of contraceptive visits from adolescents aged 10–19 years Number of schools visited by health workers for health talks Number of adolescent health-centered outreaches conducted
**Ministry of Education**	Number of pregnancy-related school dropouts Percentage of school dropouts due to adolescent pregnancy Number of non-pregnancy-related school dropouts Number and proportion of adolescents who are pregnant and have been retained in schools Number and proportion of adolescent mothers who have resumed school after pregnancy
**Ministry of Interior and Coordination**	Number of early child marriages among adolescents aged 10–19 years reported Number of household visits made to adolescents who have dropped out of school Number of cases related to adolescent pregnancies reported to police
**Multisectoral Coordination**	Number of multisectoral task force meetings held, disaggregated by sectoral participation Number of performance review meetings held Number of action points tracked Resources mobilized by the task force Number of activities jointly implemented

Upon pretesting and validating the tool, the multisectoral task forces oriented their respective officers mandated to provide data. To ensure the data used were of high quality, implementing partners supported the counties' departments of health and other sectors, including the Ministry of Education, to strengthen data quality by conducting activities such as data quality audits and supportive supervision. For example, before the data investment, reporting on the proportion of adolescents among pregnant women seeking ANC services was not standardized (e.g., some facilities counted every visit, and others restricted the count to the first ANC visit). Each sector also incorporated data privacy measures to ensure that the personally identifiable data were not being shared within the broader working group.

During quarterly performance review meetings, each sector provided data on adolescent pregnancy and other related indicators using the multisectoral tool and provided an update on progress on implementation of the action plans. The action plans would then be updated with progress made, or lack of it, and emerging issues. Through these forums, geographical areas with the highest burden of adolescent pregnancy were identified, leading to targeted prioritization of activities. After each performance review meeting, action plans were reviewed and updated with clear roles and responsibilities for each entity. These changes were implemented in the 5 scale-up counties and 3 initial counties, which adapted their models to include multisectoral monitoring.

#### National Scale-Up

Scale-up of the multisectoral approach to address adolescent pregnancy at the national level was triggered by several factors. One key platform that catapulted this approach nationally occurred during a December 2019 reproductive health meeting organized by the national Ministry of Health with participation from all 47 counties. Kwale and Homa Bay counties were requested to share their experiences in addressing adolescent pregnancies. After their presentation, the chair of the task force on adolescent pregnancy made a rallying call for all counties to adopt the Kwale multisectoral model to address adolescent pregnancy. The implementation in the 3 initial counties was so successful that the national Ministry of Health invited the county first ladies to share their learnings during the national maternal and child health stocktaking meeting—a forum that brought together the health leadership from the 47 counties.

In a separate forum, the initiative supported the National Council for Population and Development (NCPD), National HIV and STI Programme, and Ministry of Health to convene a joint meeting to discuss the burden of adolescent pregnancy, gender-based violence, and HIV/AIDS. In this forum, which was also attended by the top leadership for the Ministry of Interior and Coordination, Narok County shared its experience implementing a multisectoral response on adolescent pregnancy. Building on the momentum around data use and the formation of the multisectoral task force, the Cabinet Secretary for the Ministry of Interior and Coordination of National Government in Kenya ordered the establishment of a common reporting platform for county commissioners and regional coordinators.

All these events were happening concurrently with the ongoing related discussion at the Ministry of Health. During the same period—which coincided with the timing of the national examination of students—the media, including the AFP-trained journalists, highlighted cases of pregnant adolescent students taking the national examination. The Ministry of Education at the county level had to make special arrangements to ensure students at health facilities who were due to give birth and were still interested in taking the exams could do so. The airing of those stories and photos of adolescent girls who were pregnant or new mothers taking exams while on a maternity ward sparked a national conversation on the high burden of adolescent pregnancy. This prompted the Cabinet Secretary in Charge of Education to direct the establishment of a national task force on adolescent pregnancy. It is worth noting that, at this time, the 3 key national ministries—health, interior and coordination, and education—were giving policy direction to their respective ministries with limited interministerial coordination.

During the ICPD25+ event that was held in Nairobi in December 2019, Kenya's then president, Uhuru Kenyatta, made a country commitment to eliminate adolescent pregnancies by 2030.^21^ Consequently, the national government established an interministerial national steering committee with the NCPD as secretariat. In the national steering committee, a technical working group was formed, which resulted in the development of the *National Plan of Action for Addressing Adolescent Health Teenage Pregnancy in Kenya*.^22^ Drawing from experiences of county-level multisectoral approaches for adolescent pregnancy and the rationale that issues of adolescent pregnancy cut across various sectors, a key recommendation in the national action plan was the establishment of a county multisectoral task force chaired by county commissioners with the NCPD as the secretariat. Accordingly, the national interministerial committee, through the Ministry of Interior, directed county commissioners to implement this recommendation.

### Impact

**FIGURE 2:**
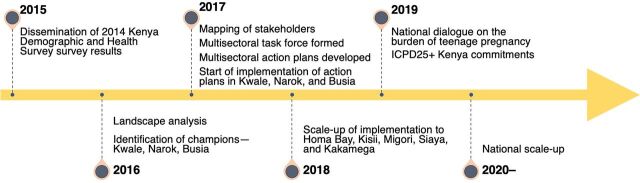
Implementation Timeline for Multisectoral Intervention in Kenya Abbreviation: ICPD, International Conference on Population and Development.

Even though it is too early to demonstrate the sustainability of the multisectoral approach that was implemented in 2016 across the country, some promising signs have been observed. Due to the national guidance from the topmost leadership, all key stakeholders, including counties and implementing partners, are pivoting their approach to be in line with the government directive. From the roll-out of the intervention up to August 2023, 38 counties of 47 had set up multisectoral action task forces, which were in different stages of formation and operation (Figure 2).

**FIGURE 3:**
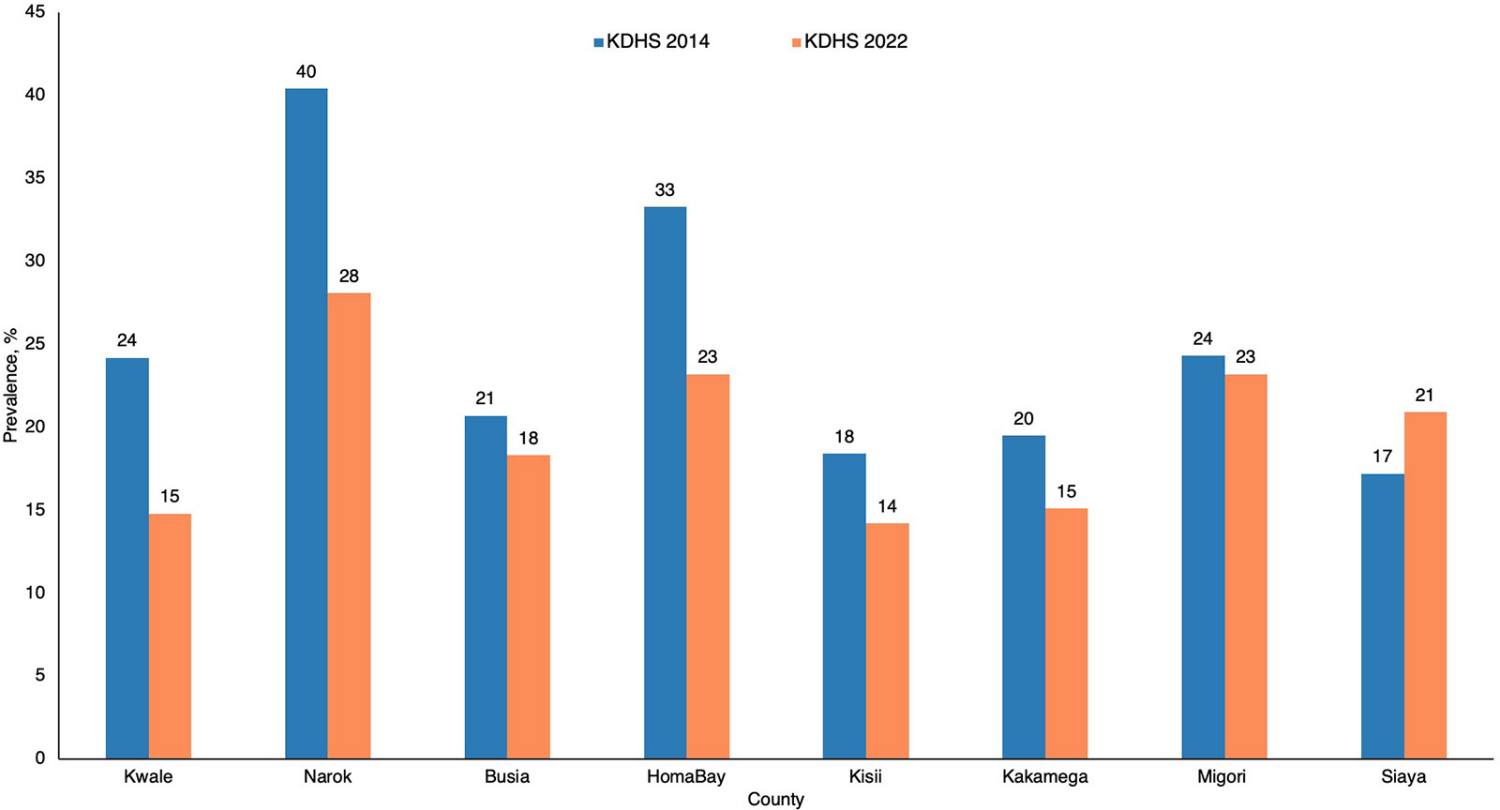
Comparison of Prevalence of Adolescent Pregnancy in Kenya Reported in KDHS 2014^a^ and 2022, by County Abbreviation: KDHS, Kenya Demographic and Health Survey. ^a^ Adolescent pregnancy prevalence reported in KDHS 2014 underestimates the magnitude as it doesn't include pregnancy losses (e.g., stillbirth and miscarriage).

The recently released 2022 Kenya Demographic and Health Survey showed a decrease in adolescent pregnancy in these counties (Figure 3).^23^ Of the 8 counties, 7 reported a reduction in adolescent pregnancy, which might be a reflection of county intervention efforts. Kwale, the first county to implement the multisectoral approach, reduced its prevalence of adolescent pregnancy by 9 percentage points.^23^ Narok County, which had the highest rates countrywide in the 2014 survey, had a reduction of 12 percentage points.^18^^,^^23^

Of the 8 counties, 7 have reported a reduction in adolescent pregnancy, which could reflect their intervention efforts.

## LESSONS LEARNED

Our reflections on key considerations for successful implementation of a multisectoral approach to address adolescent pregnancy include the following factors.

### Localization of Adolescent Pregnancy Data Localizes Challenges and Triggers Action

Decision-makers at the local level appreciate local data on the magnitude of adolescent pregnancy specific to areas of their jurisdiction. Having these data also allows advocates to frame targeted, evidence-based messaging to the decision-makers. National data are only local for decision-makers at the national level. The national data also mask subnational disparities and can create an impression, especially for subnational decision-makers, that the burden of adolescent pregnancy lies elsewhere. Moreover, existing national data on adolescent pregnancy are often not current, as the data tend to rely on the 5-year Demographic and Health Survey or the 10-year census data. This is further compounded by how the national survey data for adolescents are presented—using indicators that may not be well understood and interpreted by local decision-makers. The availability of disaggregated data from national to subnational and even the lowest level (community) allows and triggers action at various levels of decision-making.^24^

Localization of data is equally important to trigger action across the sectors. For example, the number of adolescents presenting with pregnancy at ANC was seen as data from the Department of Health, but the same number of adolescents presenting with pregnancies in schools, including school dropouts due to pregnancies, was seen as Ministry of Education data, which triggered action from the education sector. When engaging with several independent sectors to address a common problem, a common framework of measurement is needed. In our case, this was achieved through the development of the multisectoral measurement framework that guided the indicator tools to be used in tracking performance.

### Timely Media Engagement Seizing National Consciousness Generates National Conversation

The media has been a critical actor in advancing social or public policy initiatives.^25^ In Kenya, the media shaped the national conversation on ASRH by demonstrating the impact of adolescent pregnancy. While the media often told adolescent pregnancy stories in Kenya, the timing and choice of such stories were likely to determine whether there would be any follow-on actions by decision-makers. For example, when AFP supported trained journalists to access the latest data and research on ASRH, the journalists produced stories about pregnant schoolgirls during Kenya's national examinations, highlighting many cases of girls around the country who were taking their exams at the hospital before or immediately after delivery.^20^ This generated discussion on social media and several panel discussions and interviews on mainstream broadcast media on ways to address the burden of adolescent pregnancy. Consequently, the Minister of Education called for concerted efforts by actors across the sector to jointly address adolescent pregnancy across the country. This call further strengthened ongoing efforts by the 3 counties that had already established their multisectoral task forces. At the same time, the call directed the leadership of the Ministry of Education at the subnational level to take a more active role in addressing adolescent pregnancy in collaboration with other stakeholders.

### Ownership of Multisectoral Initiatives Requires a Commitment by High-Level Leadership

Successful implementation of the multisectoral approach requires ownership by all stakeholders,^10^ which requires commitment among high-level leadership at national and subnational levels. At the subnational level in the 3 counties, this commitment was realized through sectoral leadership by county commissioners and heads of the ministries of education, health, youth, and gender. These efforts gained impetus in 2019 when President Kenyatta made a commitment to address adolescent pregnancy.^21^ In his proclamation, the president directed the NCPD to spearhead all multisectoral initiatives, which fostered momentum in the counties that adopted the multisectoral approach. Counties were able to implement the approach by working closely with the NCPD county coordinators, bolstering partner commitment to support the government to implement the approach.

### Subnational-Level Interventions Can Help Trigger National Scale-Up and Inform Strategic Direction

The 3 counties used various national platforms to share their experiences implementing multisectoral initiatives. Following the mandate for NCPD to coordinate a multisectoral initiative on adolescent pregnancy across the country, benchmarking was done in those counties where key learnings were shared. The national task force officially adopted this approach and developed a national action plan to be implemented across the country, borrowing heavily from the 3 counties' multisectoral action plans. This is an example of how 3 counties in various parts of the country can inform the national-level strategic direction on addressing adolescent pregnancy. Kenya is a devolved country where the national level provides the policy and strategic guidance on operationalizing its policies, while the implementation occurs at the subnational level. However, in this case, the 3 counties shaped the policy direction of addressing adolescent pregnancy.

The national task force borrowed heavily from the 3 counties' multisectoral action plans to develop a national action plan, demonstrating how 3 counties can inform the national-level strategic direction on addressing adolescent pregnancy.

### Aligning Multisectoral Initiatives With Government Structures Enhances Their Sustainability

Anchoring multisectoral platforms under the oversight of government structures whose leadership is shielded from transitions due to political elections contributes to sustainability. The development of an action plan that speaks to the needs and aspirations of stakeholders kept the different partners engaged and helped to leverage resources and strengths to sustain a common course. Initially, the county first ladies played a key role in bringing together the various sectors. The national government's decision to have the local representatives of the NCPD serve as a secretariat and the Ministry of Interior to chair the platforms promoted sustainability.

### A Multisectoral Action Plan That Captures the Strategic Interests of Key Stakeholders Fosters Inclusivity and Continued Partnership

Each county's task force developed an action plan on adolescent pregnancy to address the fragmented implementation of programs and duplication of interventions and to leverage resources. Multisectoral task forces require deliberate collaboration and coordination among the various stakeholders and sectors for effective leveraging of resources, including the expertise to achieve a desired outcome. A key learning is that an action plan allows consensus on priorities while maintaining the strategic interests of all stakeholders. Every sector contributes its ideas, and through discussion and negotiation, critical actions and proposed activities are identified. Therefore, an action plan is a strategic tool that ensures transparency and inclusivity and provides an opportunity for effective collaboration and concerted action by different stakeholders. It helps align partners' actions and each sector's efforts to avoid duplication and inefficiencies and to facilitate collaboration. Joint development of action plans ensures that the various sectors are communicating with each other and that experiences and best practices are shared. Action plans also form a basis for reviewing progress during performance review meetings. Where different sectors fail to meet their commitments, the plan can be used as an accountability tool.

### Implementation Through an Adolescent Pregnancy Lens Provides a Less Controversial Entry Point to Broader ASRH Interventions

Access to contraceptive services by adolescents is impeded by social norms,^26^ which prevent adolescents from having the agency and autonomy to make decisions on use of ASRH services such as contraceptives. In Kenya, conversations regarding ASRH and rights are often controversial and contentious, as is the case in other African countries.^27^ For example, access to contraception among adolescents without parental consent often attracts conflicting views.^27^^–^^29^ For the multisectoral action in Kenya, using adolescent pregnancy as an entry point to address ASRH promoted buy-in of a wider stakeholder group. Focusing on adolescent pregnancy allows groups with differing views on contraceptive use by adolescents to work together. Entities opposed to adolescents accessing contraceptives will feel more comfortable being associated with activities and interventions for addressing adolescent pregnancies, as opposed to framing conversations around ASRH. It also enables opportunities for those working in related areas, such as HIV, gender-based violence, and child protection, to be part of this conversation that aims at addressing the plight of young people in totality.

### Consistency of Representation Is Key to the Effectiveness of Multisectoral Task Forces

Lack of consistency in membership on a multisectoral task force drags on the speed at which joint action plans are implemented and affects consistency of thought leadership. For example, in Siaya County, which did not show a positive trend in the prevalence of adolescent pregnancy (Figure 3), the frequent transitions during the implementation period proved to be a challenge because each new official needed to be oriented to understand the purpose and the importance of multisectoral collaboration. For effective engagement, it is imperative that a designated official from the key departments be identified through an official process to become a member of the task force—a role that is not transferable unless the transition is formally communicated. This will ensure continuity of discussions and follow-up of action plans. When transitions occur, formal communication about the transition and onboarding of the incoming official is necessary to prevent the loss of gains made. Nevertheless, transitions need not be a challenge overall, especially if the task force member is moved to another region in the same capacity. In our case, there were instances of transitions from counties implementing the multisectoral approach to other counties, which catalyzed the formation of the approach in the new counties.

## CONCLUSIONS

Our experience to date demonstrates that a multisectoral initiative can be rolled out to address issues around adolescent pregnancies in countries that continue to have a high burden of adolescent pregnancy. Successful implementation requires inclusivity, commitment, and leadership to fully realize the potential that multisectoral actions have in preventing pregnancies in adolescent girls and, as a result, improving their prospects for better health and well-being. Even though the multisectoral task force developed indicators for each sector for monitoring purposes, this was limited to what they were able to collect using their existing system. There is a need for harmonized indicators that can be used by the various sectors that traditionally do not track ASRH-related indicators.

An overarching, government-led multisectoral strategy on ASRH issues is highly beneficial as it allows multiple sectors to work synergistically toward common goals. It also helps to ensure better coordination, avoids duplication of effort, and provides a clear framework for implementing interventions across sectors and measuring progress.

Nevertheless, while a government-led multisectoral approach can bolster support for the implementation of adolescent pregnancy interventions through deliberate collaboration among various stakeholders and sectors, community-level interventions are also critical in addressing early pregnancy. ASRH still elicits a lot of interest because of the community's strong opinions on adolescent sexuality.^26^ Adolescents continue to face challenges in accessing sexual and reproductive health services, such as contraceptives, because they lack the agency, autonomy, and resources to use them.^26^ As such, social behavior change interventions that are geared toward shifting social norms should be implemented alongside the multisectoral response.

## Notes

**Peer Reviewed**

**First published online:** September 15, 2023.
